# Unmasking Rheumatic Heart Disease in Pregnancy

**DOI:** 10.7759/cureus.100328

**Published:** 2025-12-29

**Authors:** Varshitha Tumkur Panduranga, Asher Gorantla, Perman Pandal, Srivane Richard, Adam S Budzikowski

**Affiliations:** 1 Internal Medicine, State University of New York Downstate Health Sciences University, Brooklyn, USA; 2 Cardiology, State University of New York Downstate Health Sciences University, Brooklyn, USA

**Keywords:** balloon mitral valvuloplasty, echocardiography, maternal–fetal outcomes, pregnancy, pulmonary edema, rheumatic mitral stenosis

## Abstract

Pregnancy imposes significant hemodynamic stress that can unmask underlying cardiac disease. In women with rheumatic mitral stenosis, increased plasma volume and cardiac output may precipitate pulmonary congestion and heart failure, posing substantial maternal and fetal risk. A 38-year-old woman at 24 weeks of gestation presented with one week of progressive dyspnea and orthopnea, prompting suspicion for a cardiac etiology. Examination revealed tachycardia, hypertension, and a diastolic murmur at the apex. Transthoracic echocardiography demonstrated severe rheumatic mitral stenosis (mitral valve area = 0.9 cm², mean gradient = 9-10 mmHg), confirmed on transesophageal echocardiography with no left atrial thrombus. Despite medical stabilization with beta blockers and diuretics, symptoms persisted. Percutaneous balloon mitral valvuloplasty was favored over surgical intervention due to suitable valve anatomy and to avoid cardiopulmonary bypass, which carries a higher fetal loss risk in pregnancy. The procedure was performed with multidisciplinary coordination involving cardiology and maternal-fetal medicine, with continuous fetal monitoring. Following successful dilation with a 28-mm Inoue balloon, the mitral valve area increased to 1.9 cm², with rapid resolution of pulmonary edema. Both maternal and fetal outcomes were favorable at discharge.

## Introduction

Pregnancy is associated with marked cardiovascular adaptations, including increased cardiac output, plasma volume, and heart rate, which may unmask previously compensated valvular disease [1]. In women with rheumatic mitral stenosis, these physiologic changes can precipitate pulmonary edema and heart failure, conditions associated with increased maternal morbidity and adverse fetal outcomes, including preterm delivery, fetal growth restriction, and fetal loss [1]. Early differentiation from pregnancy-related hypertensive disorders, such as pre-eclampsia, is essential, as management strategies differ substantially [1]. Timely echocardiographic evaluation and multidisciplinary care are critical to guide intervention, with percutaneous balloon mitral valvuloplasty in the second trimester offering symptomatic relief while minimizing maternal and fetal risk when medical therapy is insufficient [1].

## Case presentation

A 38-year-old gravida 2 para 1 woman at 24 weeks of gestation presented to the emergency department with one week of progressive shortness of breath and orthopnea. She denied chest pain, palpitations, syncope, fever, or hemoptysis. Over the preceding two days, her dyspnea worsened significantly, limiting her ability to lie flat or perform routine activities. On arrival, she appeared in acute respiratory distress, sitting upright and tachypneic. Vital signs revealed a blood pressure of 170/110 mmHg, heart rate of 102 bpm, respiratory rate of 28/min, oxygen saturation of 88% on room air, and temperature of 36.8°C. Cardiovascular examination revealed a low-pitched diastolic murmur best heard at the cardiac apex, radiating to the axilla. Bibasilar crackles were noted on lung auscultation, with mild pedal edema and elevated jugular venous pressure. Fetal movements were present, and the uterine size was appropriate for gestational age.

The patient had no prior diagnosis of cardiac disease, hypertension, diabetes, or thyroid disorder. She denied rheumatic fever in childhood. Her obstetric history included one previous term vaginal delivery without complications. The current pregnancy had been uneventful until this presentation, with normal first and second trimester scans. She had no history of pre-eclampsia, and routine prenatal laboratory results were within normal limits.

**Figure 1:**
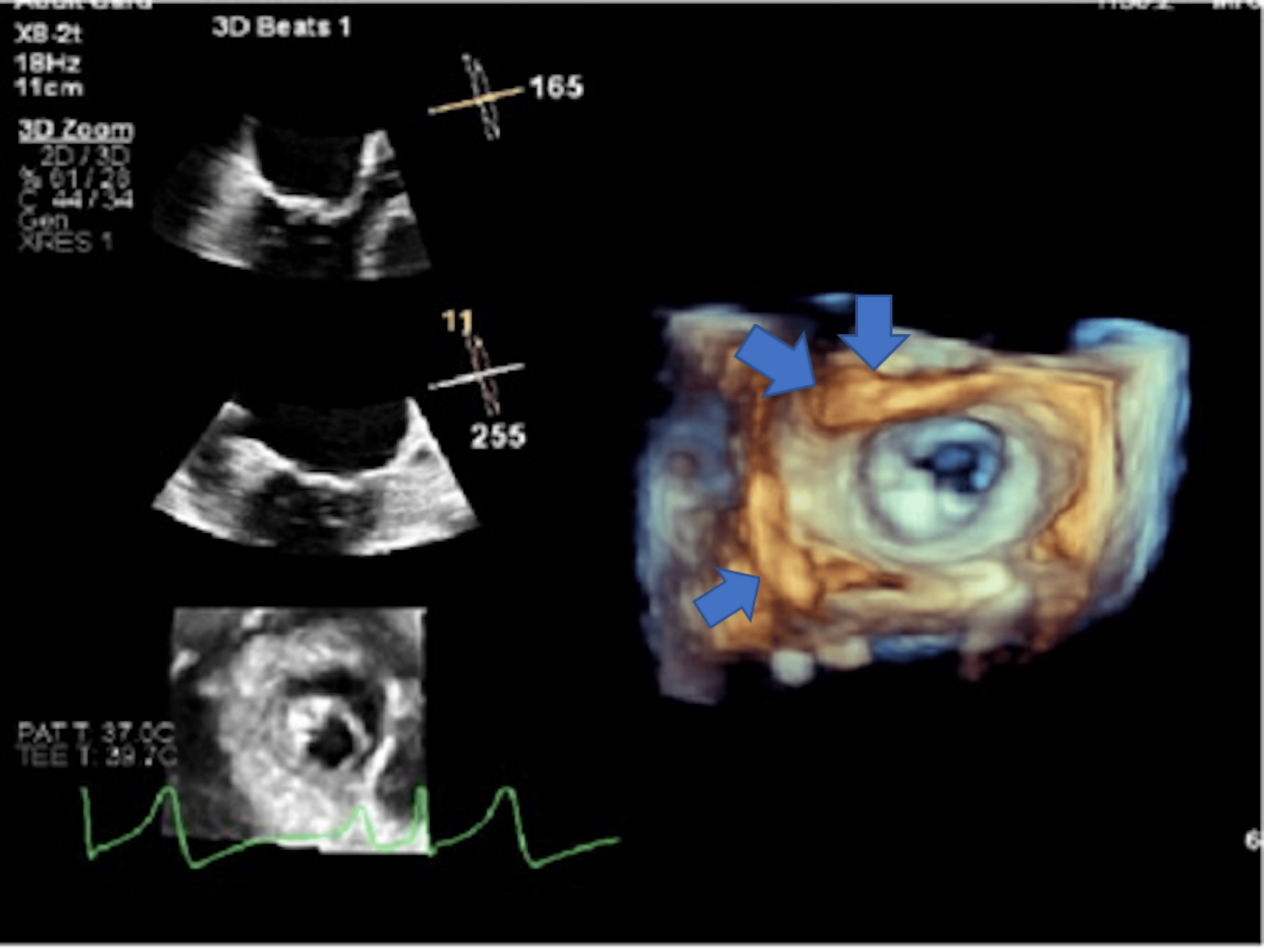
Transesophageal echocardiogram showing severe mitral stenosis with thickening of the mitral valve leaflets (blue arrows) (posterior > anterior leaflet) with a mitral valve area of 0.9 cm².

Initial laboratory studies (Table 1) revealed normal blood counts, electrolytes, and renal and liver function, with an elevated n-terminal pro-B-type natriuretic peptide level of 720 pg/mL (reference range <100 pg/mL), and negative cardiac enzymes. Urinalysis showed no proteinuria, and thyroid function was normal. Chest X-ray demonstrated pulmonary vascular congestion and mild cardiomegaly, while ECG showed sinus tachycardia with left atrial enlargement but no ischemic changes. Transthoracic echocardiography revealed severe rheumatic mitral stenosis with thickened, doming leaflets, a mitral valve area of 0.9 cm², and a mean diastolic gradient of 9-10 mmHg. Transesophageal echocardiography (Figure 1) confirmed these findings, showing no left atrial thrombus, preserved left ventricular systolic function, and mild pulmonary hypertension, establishing severe rheumatic mitral stenosis as the etiology of her acute pulmonary edema.

**Table 1:** Laboratory results. WBC: white blood cell count; BUN: blood urea nitrogen; AST: aspartate aminotransferase; ALT: alanine aminotransferase.

Test	Value	Reference range
Hemoglobin	12.8 g/dL	12–16 g/dL
Hematocrit	38%	36–46%
WBC	7.2 ×10⁹/L	4–11 ×10⁹/L
Platelets	250 ×10⁹/L	150–400 ×10⁹/L
Sodium	138 mmol/L	135–145 mmol/L
Potassium	4.1 mmol/L	3.5–5.0 mmol/L
Chloride	102 mmol/L	98–107 mmol/L
Bicarbonate	24 mmol/L	22–28 mmol/L
BUN	12 mg/dL	7–20 mg/dL
Creatinine	0.8 mg/dL	0.6–1.1 mg/dL
AST	22 U/L	10–40 U/L
ALT	18 U/L	7–56 U/L
Alkaline phosphatase	90 U/L	44–147 U/L
Total bilirubin	0.6 mg/dL	0.1–1.2 mg/dL

The patient was admitted to the cardiac intensive care unit for stabilization. Oxygen supplementation and intravenous furosemide were initiated to relieve pulmonary congestion. Metoprolol tartrate was started to control heart rate and prolong diastolic filling. Her blood pressure was cautiously managed to maintain uteroplacental perfusion. Despite medical therapy, her dyspnea persisted, and pulmonary congestion was refractory. A multidisciplinary conference involving cardiology, maternal-fetal medicine, obstetrics, and anesthesia was held. Given her severe symptomatic stenosis and risk of maternal decompensation, percutaneous balloon mitral valvuloplasty (BMV) was recommended.

**Figure 2:**
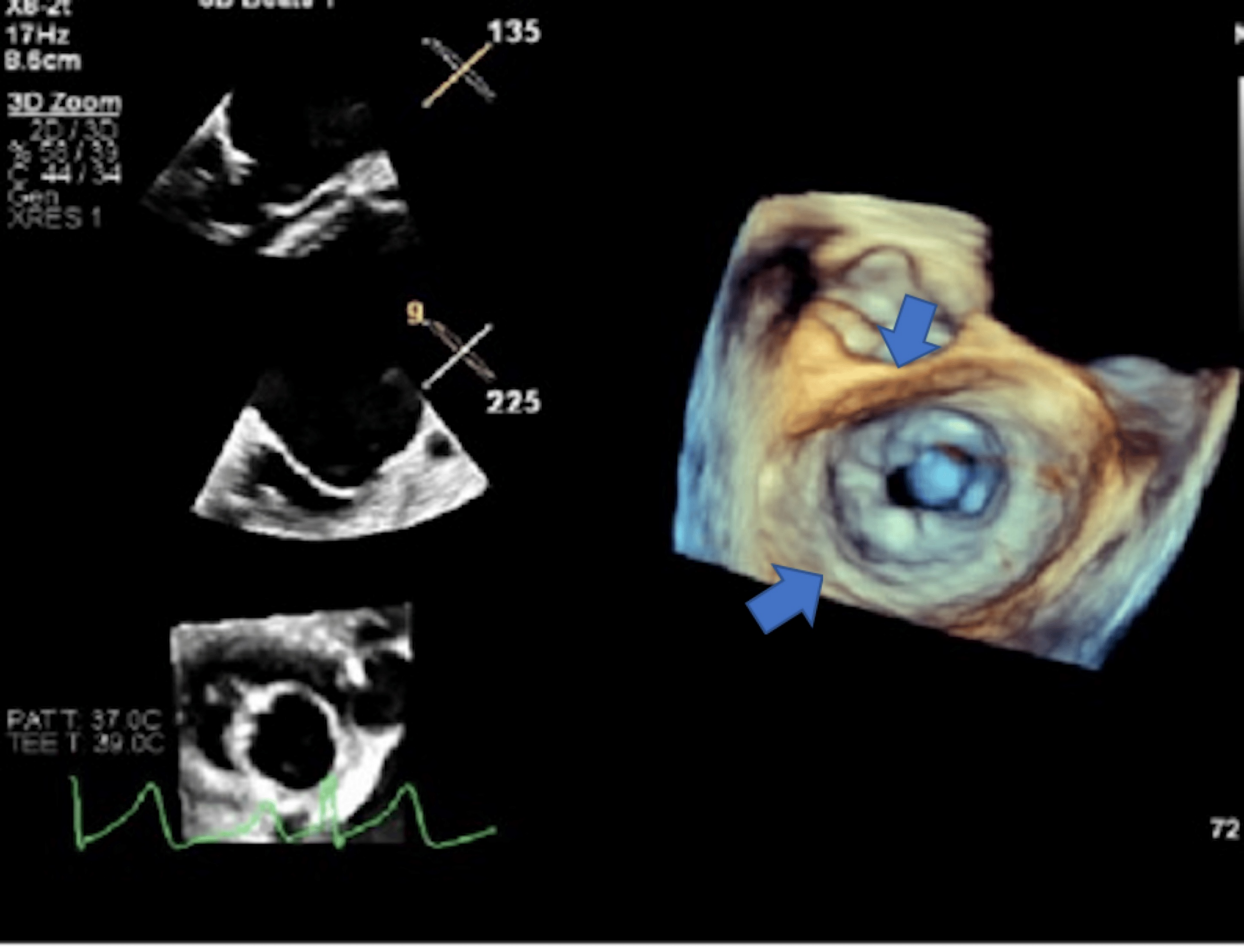
Transesophageal echocardiogram post mitral balloon valvuloplasty showing improvement in mitral valve area (blue arrows) to 1.9 cm².

Patient underwent BMV using a 28 mm Inoue balloon under fluoroscopic and echocardiographic guidance with abdominal shielding to minimize fetal radiation exposure. Post-procedure results (Figure 2) showed the mitral valve area increased from 0.9 cm² to 1.9 cm². Mean mitral gradient reduced from 9-10 mmHg to 6 mmHg, and no new mitral regurgitation or pericardial effusion was observed. Hemodynamics improved immediately, and dyspnea improved within 24 hours. Continuous fetal heart monitoring during and after the procedure showed a stable fetal heart rate pattern. Post procedure, the patient remained stable. Diuretics were tapered, and she was discharged on low-dose metoprolol and furosemide as needed, with dietary sodium restriction. The patient was scheduled with cardiology, obstetrics, and maternal-fetal medicine for closer monitoring upon discharge.

## Discussion

Mitral valve stenosis remains the most common clinically significant valvular lesion encountered during pregnancy. Although historically concentrated in low- and middle-income countries, increasing global migration has contributed to the persistence of rheumatic heart disease (RHD) in developed nations, including among women of childbearing age [1,2]. In this population, mitral stenosis is almost invariably rheumatic in origin. This case highlights how previously undiagnosed RHD may first become clinically apparent during pregnancy, when physiologic stress unmasks advanced disease.

Normal pregnancy is associated with substantial cardiovascular adaptations, including up to a 50% increase in plasma volume and a 30-50% rise in cardiac output, peaking during the second trimester [3]. In patients with mitral stenosis, the fixed obstruction to left ventricular diastolic filling results in elevated left atrial and pulmonary venous pressures, predisposing to pulmonary congestion and edema [4,5]. The risk of decompensation increases markedly when the mitral valve area is ≤1.5 cm² or when transmitral gradients rise [6]. In the present case, the patient developed acute pulmonary edema during the second trimester and was found to have severe mitral stenosis with a valve area of 0.9 cm² and a mean gradient of 9-10 mmHg, findings that directly explained her rapid clinical deterioration.

Dyspnea during pregnancy has a broad differential diagnosis, including physiologic dyspnea of pregnancy, pulmonary embolism, pneumonia, pre-eclampsia-related pulmonary edema, and peripartum cardiomyopathy. In this patient, echocardiographic evidence of severe valvular obstruction and elevated filling pressures supported a primary valvular etiology. Importantly, systolic anterior motion of the mitral valve, classically associated with hypertrophic cardiomyopathy rather than rheumatic mitral stenosis, was not a contributing factor in this case and should not be generalized to rheumatic pathology.

Current American College of Cardiology and American Heart Association guidelines emphasize that pregnancy-related hemodynamic changes can precipitate acute heart failure in women with moderate to severe mitral stenosis, particularly those with higher New York Heart Association (NYHA) functional class [7-9]. The risk of adverse maternal cardiac events rises significantly in patients presenting with NYHA class III or IV symptoms [9]. Our patient presented with NYHA class IV heart failure, a key determinant prompting escalation beyond medical therapy.

This case adds to existing evidence by illustrating how previously silent rheumatic mitral stenosis can present abruptly with life-threatening decompensation during the second trimester, despite no prior cardiac history [10]. It highlights the importance of maintaining a high index of suspicion for rheumatic valvular disease in pregnant patients with new-onset dyspnea, particularly in populations affected by global migration, where traditional geographic risk assumptions may delay diagnosis [10]. The key clinical takeaway is that early echocardiographic evaluation can be lifesaving by rapidly identifying severe valvular pathology and guiding timely escalation of care [11]. This case also underscores the critical role of structured post-procedure and postpartum follow-up, as long-term outcomes in RHD are highly dependent on ongoing surveillance and continuity of care [11].

Although initial management includes heart rate control and diuresis, percutaneous balloon mitral valvuloplasty is recommended for patients who remain symptomatic despite optimized medical therapy and have favorable valve morphology [12-14]. In this case, balloon valvuloplasty was favored over continued medical therapy or surgical intervention due to persistent hemodynamic compromise and the need for rapid symptomatic relief while avoiding the maternal and fetal risks associated with cardiopulmonary bypass.

## Conclusions

This case adds to the existing evidence by illustrating how previously silent rheumatic mitral stenosis can present abruptly with life-threatening decompensation during the second trimester, despite no prior cardiac history. It highlights the importance of maintaining a high index of suspicion for rheumatic valvular disease in pregnant patients with new-onset dyspnea, particularly in populations affected by global migration, where traditional geographic risk assumptions may delay diagnosis. The key clinical takeaway is that early echocardiographic evaluation and timely percutaneous balloon mitral valvuloplasty can rapidly stabilize both maternal and fetal status when medical therapy fails. This case also underscores the critical role of structured post-procedure and postpartum follow-up, as long-term outcomes in RHD are highly dependent on ongoing surveillance and continuity of care. From a public health perspective, it reinforces the need for improved cardiovascular screening strategies in women of childbearing age from RHD-endemic regions to prevent late presentation during pregnancy.
